# Combining Different mRNA Capture Methods to Analyze the Transcriptome: Analysis of the *Xenopus laevis* Transcriptome

**DOI:** 10.1371/journal.pone.0077700

**Published:** 2013-10-15

**Authors:** Michael D. Blower, Ashwini Jambhekar, Dianne S. Schwarz, James A. Toombs

**Affiliations:** 1 Department of Molecular Biology Massachusetts General Hospital, Boston, Massachusetts, United States of America; 2 Department of Genetics, Harvard Medical School, Boston, Massachusetts, United States of America; The John Curtin School of Medical Research, Australia

## Abstract

mRNA sequencing (mRNA-seq) is a commonly used technique to survey gene expression from organisms with fully sequenced genomes. Successful mRNA-seq requires purification of mRNA away from the much more abundant ribosomal RNA, which is typically accomplished by oligo-dT selection. However, mRNAs with short poly-A tails are captured poorly by oligo-dT based methods. We demonstrate that combining mRNA capture via oligo-dT with mRNA capture by the 5’ 7-methyl guanosine cap provides a more complete view of the transcriptome and can be used to assay changes in mRNA poly-A tail length on a genome-wide scale. We also show that using mRNA-seq reads from both capture methods as input for *de novo* assemblers provides a more complete reconstruction of the transcriptome than either method used alone. We apply these methods of mRNA capture and *de novo* assembly to the transcriptome of *Xenopus laevis*, a well-studied frog that currently lacks a finished sequenced genome, to discover transcript sequences for thousands of mRNAs that are currently absent from public databases. The methods we describe here will be broadly applicable to many organisms and will provide insight into the transcriptomes of organisms with sequenced and unsequenced genomes.

## Introduction

*Xenopus laevis* and *Xenopus tropicalis* are important model organisms for the study of developmental biology and cell cycle control[1]. For an organism to be a widely applicable model system there must be a battery of tools and resources available to the community. In addition to discipline-specific tools it is necessary to have broad community resources that aid all researchers studying an organism. One of the most important resources for any model organism community is a high-quality, well-annotated genome sequence. In 2010 the genome sequence of *Xenopus tropicalis* was published[2], which provided a tremendous improvement in the genomic resources available to the community. However, for many experiments (cell cycle extracts and many developmental techniques) *Xenopus laevis* remains the preferred (or exclusive) frog of choice[1] and the genome sequence of *X. tropicalis* does not directly aid *X. laevis* research. Genome sequencing of *X. laevis* has proceeded at a slower pace (due partly to the fact that *X. laevis* has an allotetraploid genome compared to the diploid genome of *X. tropicalis*), but will eventually provide genomic resources to the frog community.

For the purpose of studying the function of a given protein, having a list of expressed mRNAs (transcriptome) at a given developmental time point would be an acceptable alternative to a complete genome sequence. Transcriptome data is also an essential complement to the completed genome sequence as it provides a record of which genes are expressed at given times and the precise structure of each transcript, which is information that can be difficult or impossible to determine from the genome sequence alone[3]. The *Xenopus laevis* community has invested significant time and resources into developing full-length cDNA clones and libraries of ESTs to provide insight into the frog transcriptome[4]. While great progress has been made, the current cDNA and EST sequences are likely to account for only ~50% of all mRNAs[4] and contain biases towards highly expressed genes and against very long mRNAs.

One hallmark of *Xenopus* oogenesis and early development is the use of post-transcriptional RNA modification to determine the timing of protein expression[5]. In particular, work from many different labs has shown that control of poly-A tail length can lead to translational activation or repression in the absence of changes to mRNA abundance[5,6]. Small scale studies of mRNAs whose poly-A tails change in length during oogenesis and early embryogenesis have lead to the discovery of many important proteins that control the cell cycle[7], suggesting that genome-wide approaches will likely provide additional insight into important cellular regulatory networks as has been observed in budding yeast[8].

The recent emergence of high-throughput DNA/RNA sequencing technologies (RNA-Seq) offers a viable, cost-effective manner to begin to assemble the complete transcriptomes of model organisms without fully sequenced genomes[9]. Several groups have developed *de novo* transcriptome sequence assemblers designed to work with short sequence reads produced by current high-throughput DNA sequencing platforms (reviewed in 9). Several different *de novo* assemblers have been designed to provide relatively sensitive reconstruction of transcripts with very different abundance levels, can be used in combination with a reference genome of a closely related organism to generate longer transcript fragment (trans-frag) assemblies[10,11], accurately discriminate paralogs[12] and provide annotation to the RNA sequences by homology to known genes in well-annotated organisms. 

In addition to providing a cost effective method for providing a catalog of expressed genes, RNA-seq is uniquely able to detect regulatory RNAs. Recent work in several different organisms (most notably human, mouse, and zebrafish) has shown that hundreds to thousands of RNAs are expressed with little protein coding potential[13-16]. However, because these RNAs lack protein coding potential they are missed by gene prediction algorithms, and because they are expressed at low levels, they are largely missing from cDNA and EST resources. Furthermore, many organisms express regulatory RNAs antisense to known protein coding transcripts that will be absent or missannotated in transcript prediction and annotation methods. However, because RNA-Seq can be performed in a strand specific manner it is uniquely able to capture the complexity of the transcriptome.

The first step in any study of the transcriptome is enrichment of mRNA from the vastly more abundant ribosomal RNA (rRNA). Most methods isolate mRNA by using oligo-dT to capture the mRNA through the poly-A tail. However, the efficiency of mRNA capture by oligo-dT is a function of the length of the poly-A tail, with mRNAs containing short poly-A tails being captured inefficiently[17]. In this study we tested the hypothesis that combining different mRNA capture methodologies would lead to a more complete view of the transcriptome and provide a genome-wide view of mRNAs regulated by cytoplasmic polyadenylation. We combined mRNA capture via the poly-A tail with capture via the 5’ 7-methyl guanosine cap to survey the transcriptome. We found that these approaches capture different subsets of the transcriptome and that when combined can predict mRNAs that are undergoing changes in poly-A tail length. We then used reads generated from these sequencing libraries to perform *de novo* transcriptome assembly. We find that while each mRNA capture method recovers different transcripts, the mRNAs captured by the 5’Cap provide a broader view of the transcriptome. Our results suggest that combining mRNA capture methods with RNA sequencing and *de novo* transcriptome assembly is an efficient method to gain insight into the expressed mRNAs of an organism with an unsequenced genome. 

## Results

### Comparison of different mRNA capture methods

One of the main technical problems confronting researchers when they attempt to clone or sequence mRNAs is separation of mRNA from ribosomal RNA. In most cells rRNA makes up the vast majority of the total RNA (up to 95% in the *Xenopus* egg) and if it is not removed will dominate the clones or sequences[18]. The most common method for removal of rRNA takes advantage of the fact that mRNAs generally contain a poly-A tail at the 3’ end while rRNA does not. Selective capture or priming of reverse transcription using oligo-dT is the basis for the majority of cDNA library preparations and RNA-Seq library preparations. However, many mRNAs undergo changes in poly-A tail length as a function of their normal life cycle[19] and poly-A tail length affects the efficiency mRNA recovery by oligo dT-based methods[17]. In addition to being polyadenylated all mRNAs receive a 5’ 7-methyl guanosine cap in the nucleus. The cap structure is required for the majority of mRNA translation and is bound by the translation initiation factor eIF4E[20]. To determine if an alternative method of mRNA capture could be complementary to mRNA capture by oligo-dT selection we optimized a previously described method[20,21] to use recombinant human cap-binding protein eIF4E to capture mRNAs on the basis of the 5’ cap structure. 

To compare the mRNAs sampled by oligo-dT and Cap-capture methods we prepared Illumina libraries from *Xenopus laevis* egg extracts arrested in metaphase of meiosis II (labeled Mitosis or M for the remainder of the paper) and extracts induced to enter interphase (IF) by the addition of calcium, which mimics fertilization induced calcium release[22]. We chose this system as a test case because previous work has shown that many mRNAs have different poly-A tail lengths in *Xenopus* eggs compared to *Xenopus* embryos[7]. Furthermore, mature *Xenopus* eggs and early embryos are transcriptionally silent[23,24] and have very little RNA degradation[25], therefore any observed changes in mRNA abundance are likely to be the result of changes in poly-A tail length rather than increased transcription or mRNA degradation. We used Bowtie[26] to align reads from these libraries to the NCBI Unigene *Xenopus laevis* database and *X. laevis* rRNA precursor. Since mRNA-seq libraries that are primed using random hexamers from unselected total RNA result in 80-90% of reads mapping to rRNA[18] we aligned our sequences to the *X. laevis* rRNA precursor to estimate mRNA enrichment. Consistent with previously published reports, we found that both oligo-dT and Cap-capture selections effectively removed rRNA sequences (Table 1) with less than 4% of reads aligning to rRNA. In addition, semi-quantitative PCR analysis of mRNAs purified by Cap-capture and oligo-dT demonstrate that both selection methods enrich mRNA to a similar extent (Figure S1). Both selection methods were highly reproducible when comparing technical replicates from the same extract (Table 1).

**Table 1:** Alignment characteristics of various sequencing libraries.

**mRNA capture**	**Egg: dT**	**Egg: Cap**	**Oocyte I-III: dT**	**Oocyte I-III: Cap**
**Technical Replicate R^2^**	**0.99**	**0.99**	**NA**	**NA**
**% rRNA**	**1.4**	**3.5**	**0.02**	**0.9**
**% Alignment to X.l Unigene**	**56**	**43**	**59**	**48**
**Mitosis:Interphase R^2^**	**0.97**	**0.99**	**NA**	**NA**
**% Alignment to *de novo* assembly**	**86**	**81**	**80**	**80**

**Figure 1:**
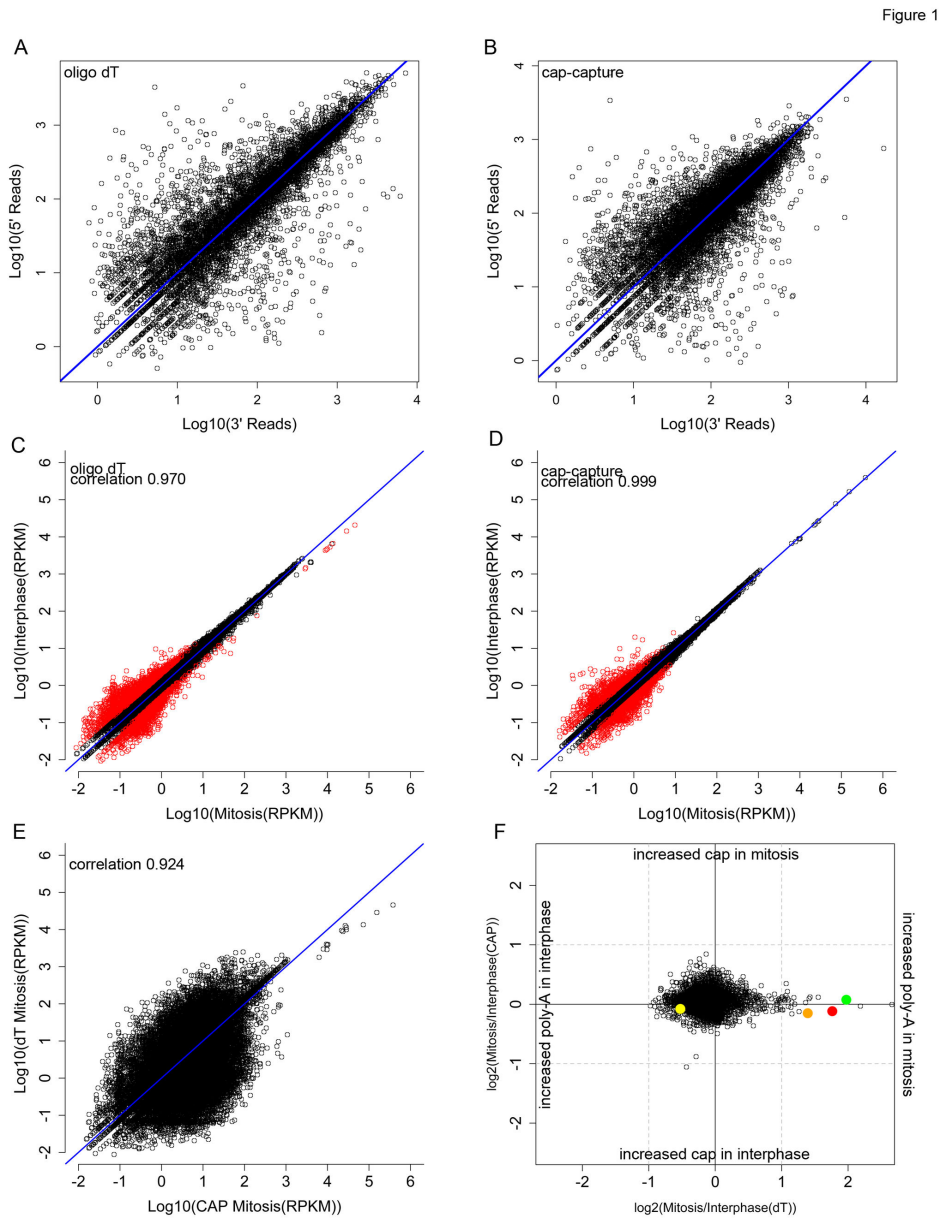
Comparison or mRNA-seq libraries from oligo-dT and Cap-captured mRNA. A-B. Reads from dT or Cap-capture prepared libraries were aligned to sequences consisting of the 5’ or 3’ 25% of Refseq mRNAs. Reads aligning to the 5’ and 3’ portions of the transcript are plotted. Blue line indicates a ratio of one. C. Reads from mRNA-seq libraries prepared using oligo-dT captured mRNAs from mitotic and interphase *Xenopus* egg extract were aligned to the *Xenopus laevis* Unigene database. Relative abundance of each mRNA in mitotic and interphase extracts is plotted. Red points highlight two-fold differences between mitotic and interphase samples. D. Same experiment as in panel C except that the mRNAs were purified using Cap-capture prior to library preparation. Red points highlight two-fold differences. E. Scatterplot of mRNA abundance in oligo-dT-captured and Cap-captured mRNA libraries. F. The ratio of reads per mRNA in mitotic and interphase extracts are plotted for oligo-dT captured mRNAs (X-axis, data from panel A) and cap-captured mRNAs (Y-axis data from panel B). The colored points correspond to mRNAs with known changes in poly-A tail length (cdk1, green; Eg2/aurora-a, orange; mos, red; Xlcl1, yellow). Quadrants highlight two-fold differences between samples.

Capturing mRNAs by the 5’ or 3’ end could result in selective recovery of mRNA fragments coming from partially degraded transcripts. To determine if there was a bias in recovery of either end of the mRNA using Cap-capture or dT selection, we compared read coverage at the ends of mRNAs. We used the well-annotated *X. laevis* Refseq transcript database because these are full-length mRNAs with known polarity. We then counted the reads that mapped to the 5’ or 3’ quartile of each transcript and compared coverage between the two ends. We found that both mRNA capture approaches resulted in nearly equal coverage at both ends of the mRNA (Figure 1A-B), demonstrating that both capture strategies recover predominately full length mRNAs. 

To determine if mRNA capture method can affect mRNA recovery, we compared normalized transcript abundance between mitotic and interphase extracts. Since these are identical extracts that differ only in the cell cycle state, we expected high correlation. Analysis of Cap-selected mRNAs showed excellent correlation between mitotic and interphase extracts with little variation in transcript abundance and essentially no variation at high expression levels (Figure 1D). In contrast, oligo-dT selected mRNAs showed some variation between mitotic and interphase extracts even at very high expression levels (Figure 1C). To determine if oligo-dT and Cap selection recover equal amounts of the same mRNAs, we compared the levels of mRNAs present in oligo-dT and Cap captured mRNA libraries. We found that there was significant variation in apparent levels of mRNAs between different mRNA selection methods, but an overall reasonable correlation (Figure 1E). To determine if the increased variation observed in oligo-dT selected libraries and differences in apparent transcript abundance between oligo-dT and Cap selected libraries could result from differences in poly-A tail length we examined apparent mRNA abundance in mitotic and interphase extracts from both selection methods for highly expressed mRNAs (>= 100 reads in mitotic extracts for both cap and dT libraries). We found that most mRNAs were equally abundant in both mitotic and interphase extracts. However, oligo-dT selected mRNAs showed a higher variation in apparent abundance between mitotic and interphase extracts (Mitosis/Interphase mean=0.96+/-0.3) while Cap-capture libraries showed less variation between the two extracts (Mitosis/Interphase mean=1.03+/- 0.17). Interestingly, when we compared the ratio of mRNA abundance in mitotic and interphase extracts between the two methods, we found that many mRNAs exhibited no abundance change in Cap captured libraries while they exhibited large changes in abundance oligo-dT captured libraries (Figure 1F). Changes in mRNA abundance in oligo-dT selected libraries but not in Cap-captured libraries suggested that the apparent changes in oligo-dT selected libraries could be a result of changes in poly-A tail length rather than changes in mRNA abundance. Consistent with this hypothesis several mRNAs that are known to be deadenylated at fertilization (Eg1(cdk1, green), Eg2(Aurora-A, orange)[7], and mos(red)[27]) exhibited a high mitosis to interphase ratio. In addition, the Xlcl1 (yellow) mRNA that is known to be polyadenylated at fertilization[7,28] showed a high interphase to mitosis ratio with no apparent change in Cap-captured mRNA abundance (See Table S2 (high M:IF) and Table S3 (low M:IF) for a complete list). 

In general the changes in apparent abundance suggested that mRNA deadenylation proceeds rapidly in *Xenopus* extracts (high M:IF ratio), but that adenylation may proceed more slowly. Although there was less variation in apparent abundance in mRNAs captured by the 5’cap there were some RNAs that showed large differences between mitosis and interphase extracts. It is possible that these mRNAs are examples of regulated decapping or recapping[29,30]. These results suggest that when these mRNA selection approaches are used in combination they can provide insight into mRNA adenylation status.

### Combined mRNA capture sequencing can identify changes in poly-A tail length

**Figure 2:**
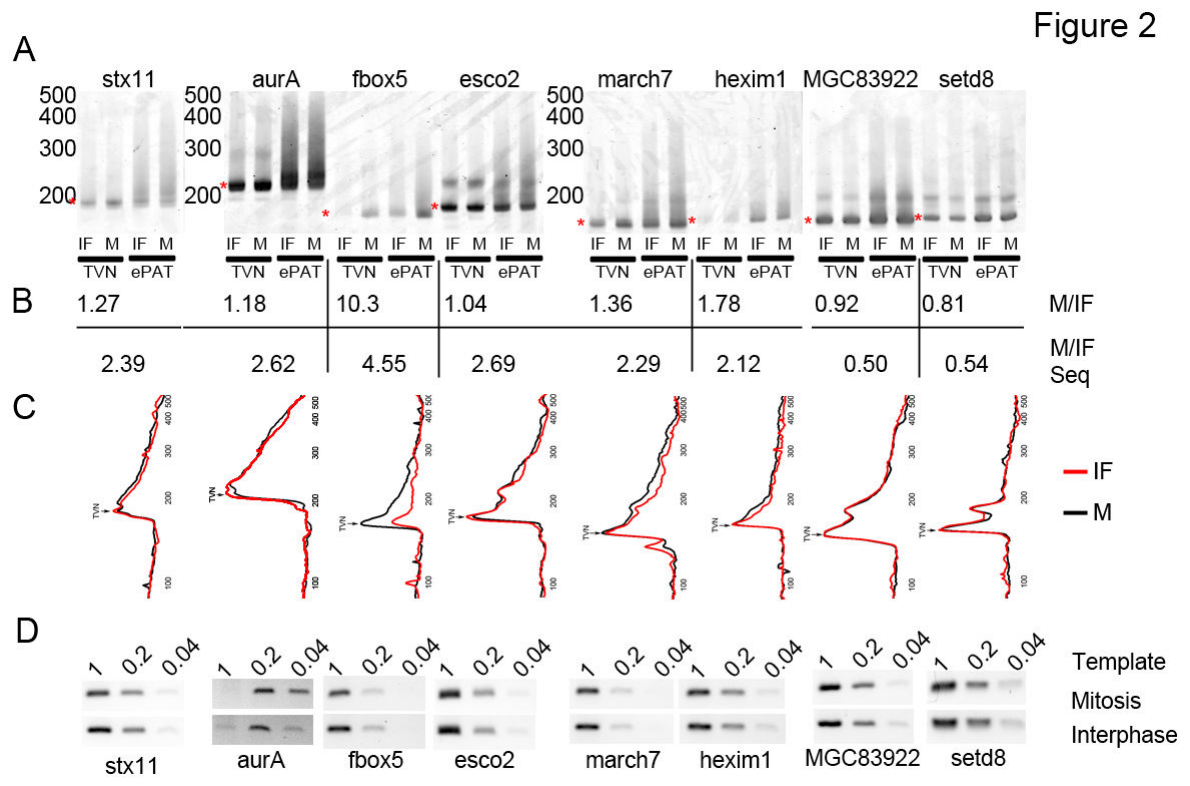
Poly-A tail analysis of selected mRNAs. mRNAs that exhibited changes in Mitosis:Interphase (M:IF) abundance ratios in oligo-dT-captured samples, but not in Cap-captured samples were analyzed for poly-A tail length using the ePAT assay and anchored TVN reverse transcription controls. A. Six mRNAs with high M:IF ratios (aurora-a, esco2, fbox5, stx11, march7, and hexim1) showed longer poly-A tails in mitotic extract. Two mRNA (setd8 and MGC83922) with a low M:IF ratio showed very modest changes in poly-A tail lengths between Mitosis and Interphase. Red asterix indicates the position of the prominent TVN PCR product that is quantified in B. B. The amount of PCR product contained in the TVN-RT PCR reactions (red asterix in A) were quantified. The ratio of the amount of PCR product in Mitotic to Interphase extracts is presented in the first line. The ratio of each mRNA in Mitotic and Interphase extracts as determined by RNA-seq is presented below the PCR derived ratios for comparison. In addition five mRNAs with high M:IF ratios (aurora-1, fbox5, stx11, march7, and hexim1) had increased amounts of minimal poly-A tail PCR products in mitosis compared to interphase in TVN controls (quantified below gel) while both setd8 and MGC88922 had higher levels of TVN PCR products in interphase compared to mitotic extracts TVN PCR products indicate mRNAs with poly-A tails of 18 As. Line traces of ePAT PCR reactions presented in panel A. Black lines indicate traces from mitotic extract and red lines indicate traces from interaphse extract. D. Semi-quantitative PCR for each of the mRNAs tested in A was performed on RNA from mitotic and interphase extracts. Random hexamers were used to prime reverse transcription for these reactions. A second experiment showing very similar results is present in Figure S2.

Changes in the apparent abundance of mRNAs selected by oligo-dT, but not by Cap-capture, may actually represent alterations in poly-A tail length. We hypothesized that two different types of changes in poly-A tail length could occur: first, the overall length of the poly-A tail could decrease dramatically (from hundreds of As to a minimal A tail) or; second, a short poly-A tail could be shortened below the length of the capture oligo. To determine if either of these types of changes occurred, we used the recently developed extension poly-A tail test (ePAT[31]) and PCR to compare poly-A tail lengths in mitotic and interphase extracts. As a control, we performed reverse transcription with an anchored oligo-dT primer (TVN) (consisting of 18Ts followed by V (A|G|C) N (A|T|G|C), which anchors the primer at the start of the poly-A tail) to mark a minimal poly-A tail (18 As). We tested the adenylation status of 6 mRNAs that appeared to be deadenylated in interphase extracts (auroraA, stx11, march7, fbox5, esco2, and hexim1) and two mRNAs that showed modestly increased adenylation in interphase extracts (setd8 and MGC83922). Using the ePAT assay and TVN control we found two subtle, but reproducible changes (Figure 2 and Figure S2) in the poly-A tails of each of the mRNAs that we tested. We found that a control mRNA that is known to be deadenylated at fertilization (aurora-A, Eg2)[7] and four of five uncharacterized mRNAs that had high mitosis:interphase ratios (fbox5, march7, stx11, and hexim1) showed longer poly-A tails in mitotic extracts compared to interphase extracts (Figure 2A and C and Figure S2). Interestingly, we also noted that each mRNA with a predicted higher polyadenylation in mitosis produced consistently more PCR product in TVN control reactions from mitotic extracts compared to interphase extrats (with the exception of esco2)(Figure 2A-B). To measure changes in the minimal poly-A tails, we quantified the amount of product in the unadenylated band position (Figure 2A red asterix). We found that mRNAs with increased adenylation at mitosis had high mitosis to interphase ratios in TVN controls (Figure 2B), consistent with increased polyadenylation during mitosis. This suggests that the poly-A tails of each of these mRNAs is reduced to less than 18 As following entry into interphase, which results in inefficient RT priming with the oligo dT primer. Deadenylation to produce very short polyA tails is consistent with a loss of signal in sequencing libraries that rely on dT hybridization to the poly-A tail. In addition, two mRNAs that exhibited a low mitosis:interphase ratio by sequencing (setd8 and MGC83922) did not exhibit dramatically longer poly-A tail in interphase extracts compared to mitotic extracts (Figure 2A-C and Figure S2). However, each of these mRNAs had a low mitosis:interphase ratio in TVN controls, consistent with a subtle lengthening of the poly-A tail during interphase. The fact that we can detect changes in mRNA recovery based on oligo-dT priming that are consistent with our sequencing results suggests that each of these mRNAs is undergoing regulated poly-A tail control at the mitosis to interphase transition. Our results are also consistent with the sequencing data in that mRNA deadenylation in interphase is more dramatic than mRNA adenylation. To confirm that the changes in mRNA abundance that we observed were the result of changes in poly-A tail length, we used random hexamers to prime reverse transcription of total RNA from mitotic and interphase extracts followed by semi-quantitative PCR for each of the mRNAs tested. We found that each mRNA was present at equal amounts in mitotic and interphase extracts (Figure 2D), consistent with the interpretation that the observed changes in mRNA recovery are the result of changes in poly-A tail length rather than changes in mRNA abundance. These results demonstrate that combining mRNA capture by oligo-dT and Cap-capture can be used to identify changes in mRNA poly-A tail length on a genome-wide scale and identify novel mRNAs undergoing poly-A tail length regulation. Furthermore, these results suggest that changes in mRNA abundance detected by methods based on oligo-dT selection may not accurately report on mRNA abundance, but rather a combination of mRNA abundance and poly-A tail length.

### Comparison of *de novo* transcriptome assembly from different mRNA pools

During the course of analyzing sequencing libraries (Table S1) generated by both oligo-dT and Cap based mRNA capture strategies, we noted that a considerably lower percentage (~10-12% less) of reads coming from Cap capture mRNA libraries aligned to the *X. laevis* Unigene database (Table 1). Since the majority of the sequences that are present in the Unigene database come from sequencing projects that use oligo-dT priming as the first step in mRNA synthesis, we hypothesized that the Unigene database might under represent mRNAs with short poly-A tails. Furthermore, the low overall rate of read alignment to the Unigene database (less than 60% of reads aligning) suggested that the Unigene database is not a complete archive of *X. laevis* transcripts. 

The recent development of *de novo* sequence assemblers that are specifically designed to assemble transcripts from short sequencing reads[9] offered the possibility to determine if different mRNA capture methodologies capture different pools of the transcriptome, and furthermore, if it is possible to assemble large fragments (or complete transcripts) that are not currently present in the *X. laevis* Unigene database. Two prominent *de novo* sequence assemblers that have performed well under a variety of experimental conditions are Velvet and Abyss[32-35]. Recent work has shown that *de novo* assemblies performed using a range of different k-mers (the length of sequence overlap between sequences to form a contig) can capture a larger number of transcripts coming from a wide range of expression levels[11,12,33,34]. A crucial step in *de novo* assembly using multiple k-mers is merging multiple assemblies to generate the longest possible transcript. This is complicated in an organism such as *X. laevis* that has a allotetraploid genome[1], which results in very closely related sequences arising from different genes as a result of genome duplication (paralogs). A previous study of full-length cDNA sequences from both *X. laevis* and *X. tropicalis* demonstrated that paralogous genes uniquely present in *X. laevis* were 93.1% +/- 2.72% identical at the nucleotide level[36]. As a result of this high sequence identity we required 100% sequence identity when we merged transcripts from different assemblies to retain sequence information about paralogous transcripts. 

To determine if oligo-dT and Cap captured mRNAs capture different pools of the transcriptome we used ~17 million unique, non-rRNA reads from both oligo-dT and Cap capture libraries as input for the *de novo* assemblers Velvet and Abyss using a range of k-mer sizes. We then combined the transcripts generated by all subassemblies for each capture method and assembled and removed all sequences completely contained in another longer sequence using BLAT and a custom Perl script. At the same time we removed all transcript sequences of less than 100nt. Each assembler and input library generated a varying number and length of transcripts (Table 2). In general, Abyss generated a smaller number of transcripts, but more long transcripts than Velvet (Table 2). Interestingly, in both assemblies, libraries generated from Cap-captured mRNAs generated a larger number of transcripts than those generated from oligo-dT captured libraries (Table 2). 

**Table 2:** *De novo* assembly characteristics.

**Library**	**dT**		**Cap**	
**Assembler**	**Abyss**	**Velvet**	**Abyss**	**Velvet**
**Sequences>100bp**	**86052**	**166333**	**156801**	**318665**
**Transcripts > 0.5Kb**	**12349**	**15874**	**16647**	**21596**
**Transcripts > 1Kb**	**3692**	**3555**	**4002**	**5622**
**Transcripts > 5 Kb**	**50**	**19**	**71**	**16**

**Figure 3:**
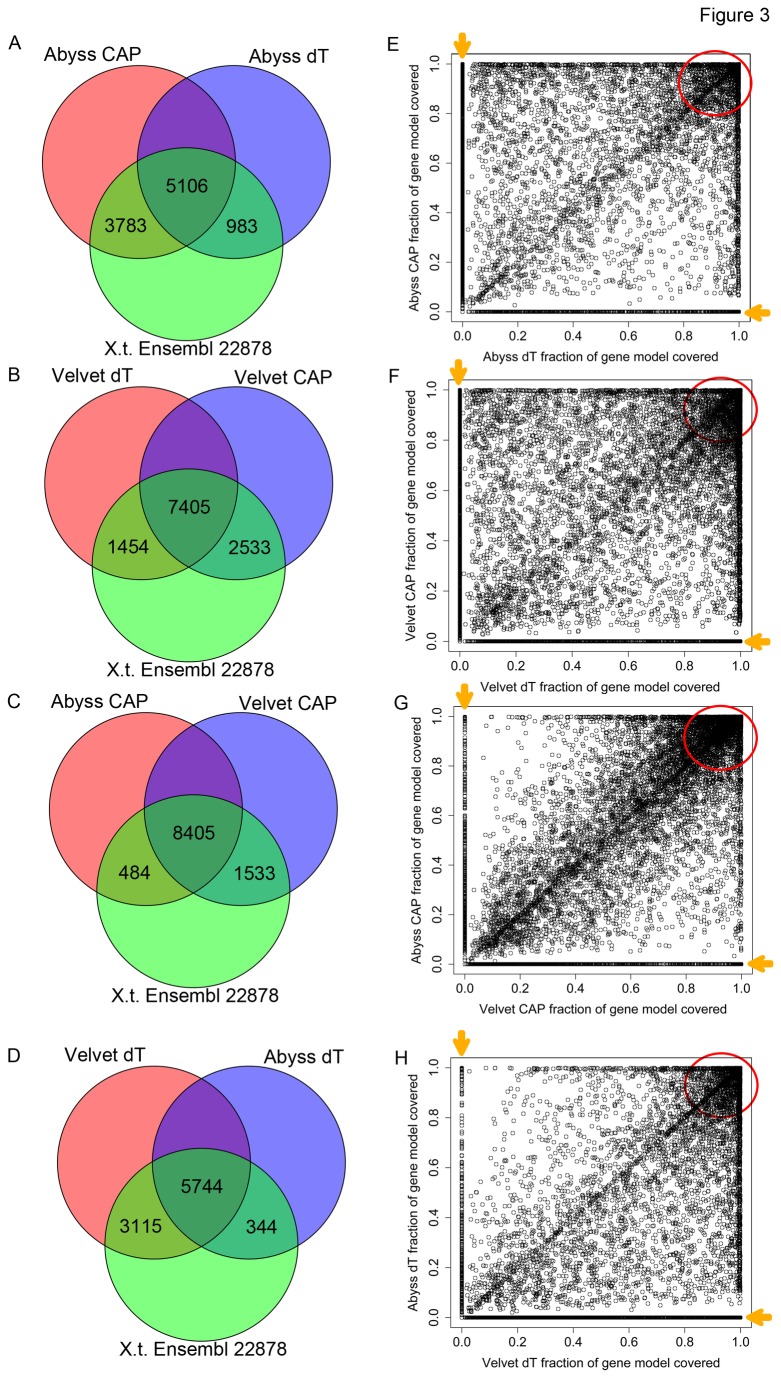
Comparison of *de* *novo* transcriptome assemblies. A. Transcripts assembled by the Abyss assembler from oligo-dT and Cap-captured mRNA libraries were aligned to the *X. tropicalis* ENSEMBL annotated transcripts using BlastX. The number of ENSEMBL transcripts that were matched by each assembler:library pair are represented as a Venn Diagram. The number of transcripts present in each assembler:library pair are listed in Table 2. Because multiple sequences from each *de* *novo* assembly align to *X. tropicalis* genes these numbers are omitted from the figure for the sake of simplicity. B. Same comparison as in A, except that Velvet was used as the assembler instead of Abyss. C. Transcripts assembled by Abyss or Velvet from Cap-capture mRNA libraries were aligned to the *X. tropicalis* ENSEMBL transcripts using BLAT. Unique and common ENSEMBL transcripts are represented by a Venn Diagram D. Same comparison as in C, except that dT libraries are compared instead of Cap-captured libraries. E-H. Fraction of each ENSEMBL transcript covered by transcripts assembled using indicated assembler:library pair (from A-D) was calculated.

To determine if our *de novo* transcript assemblies accessed different portions of the transcriptome, we aligned our transcripts to a reference transcriptome. Because there is currently no well-annotated genome sequence available for *X. laevis* we aligned our sequences to the transcriptome of the closely related *X. tropicalis*. Although there are no completely annotated transcript sets for *X. tropicalis*, we chose the ENSEMBL annotated transcript set as it was the largest and most well-annotated available. We aligned our transcripts to ENSEMBL transcripts using BlastX, requiring a minimum evalue of 1E-10. Using these criteria we, confirmed that different methods of mRNA capture samples different portions of the transcriptome (Figure 3A-D). Using both Abyss and Velvet we found that Cap captured mRNAs identified transcripts homologous to a larger number of *X. tropicalis* mRNAs (Figure 3A-B). However, we found that there was a subset of transcripts that was uniquely identified using oligo-dT capture, but not using Cap captured mRNA. Interestingly, when we compared the transcripts assembled using the same dataset but different assemblers, we found that each assembler reconstructed some unique transcripts (Figure 3D-E). In general, we found that Velvet reconstructed more transcripts than Abyss, but that a subset of transcripts was uniquely assembled using the Abyss assembler. 

To determine if identification of mRNAs using specific capture methods and *de novo* assemblers resulted from low abundance transcripts, we compared transcript coverage using our *de novo* assembled sequences. We calculated the portion of each ENSEMBL transcript that was covered by BlastX matches from *de novo* assembled sequences and compared transcript coverage between different methods. If transcripts were identified in one mRNA capture assembler pair but not another because of low abundance, we would predict that transcript coverage would be low for transcripts present in one combination but not another. Interestingly, when we compared oligo-dT and Cap-capture transcript coverage, we found that there were several interesting features of the coverage plots. First, there was a large class of mRNAs that had nearly complete coverage in both mRNA capture strategies (Figure 3E-F, red circles). Second, there were many mRNAs that were present in one strategy, but absent from another. The coverage of these mRNAs ranged from very low to complete coverage (Figure 3E-F orange arrows). Similarly, when we compared coverage generated using different assemblers, we found that there was a group of transcripts with reasonable correlation between the transcript coverage (diagonal in plot), a large group of mRNAs with complete coverage in both assemblers (red circles), and transcripts spanning the coverage spectrum that were uniquely present in one assembler but not the other (Figure 3G-H orange arrows). These results demonstrate that different mRNA capture methods identify different and unique portions of the transcriptome. Furthermore, differences in the underlying assembly methods result in differential assembly of individual transcripts. Taken together, these results suggest that each of these approaches are highly complementary to one another and can likely be combined to generate a more complete transcriptome.

The number of transcripts assembled using *de novo* assemblers is proportional to the number of reads used as input. To determine if Cap-capture libraries recovered transcripts that were present at very low levels in oligo-dT libraries, we compared the number of transcripts assembled using 17M Cap-capture reads and an increased number of oligo-dT reads (44M). We used BlastX to map the assembled transcripts to *X. tropicalis* Ensembl annotated mRNAs. We found that increasing the number of dT reads increased the number of transcripts assembled and that there was a greater overlap between dT and Cap-capture libraries (Figure S3). Interestingly, we found that there were still 1443 transcripts that were uniquely assembled using Cap-capture libraries. These results suggest that increasing the number of sequencing reads will provide greater coverage of transcripts as one would expect, and importantly, that Cap-capture more efficiently accesses a subset of the transcriptome than does poly-A selection.

### Combined assembly using pooled mRNA libraries and assemblers

**Figure 4:**
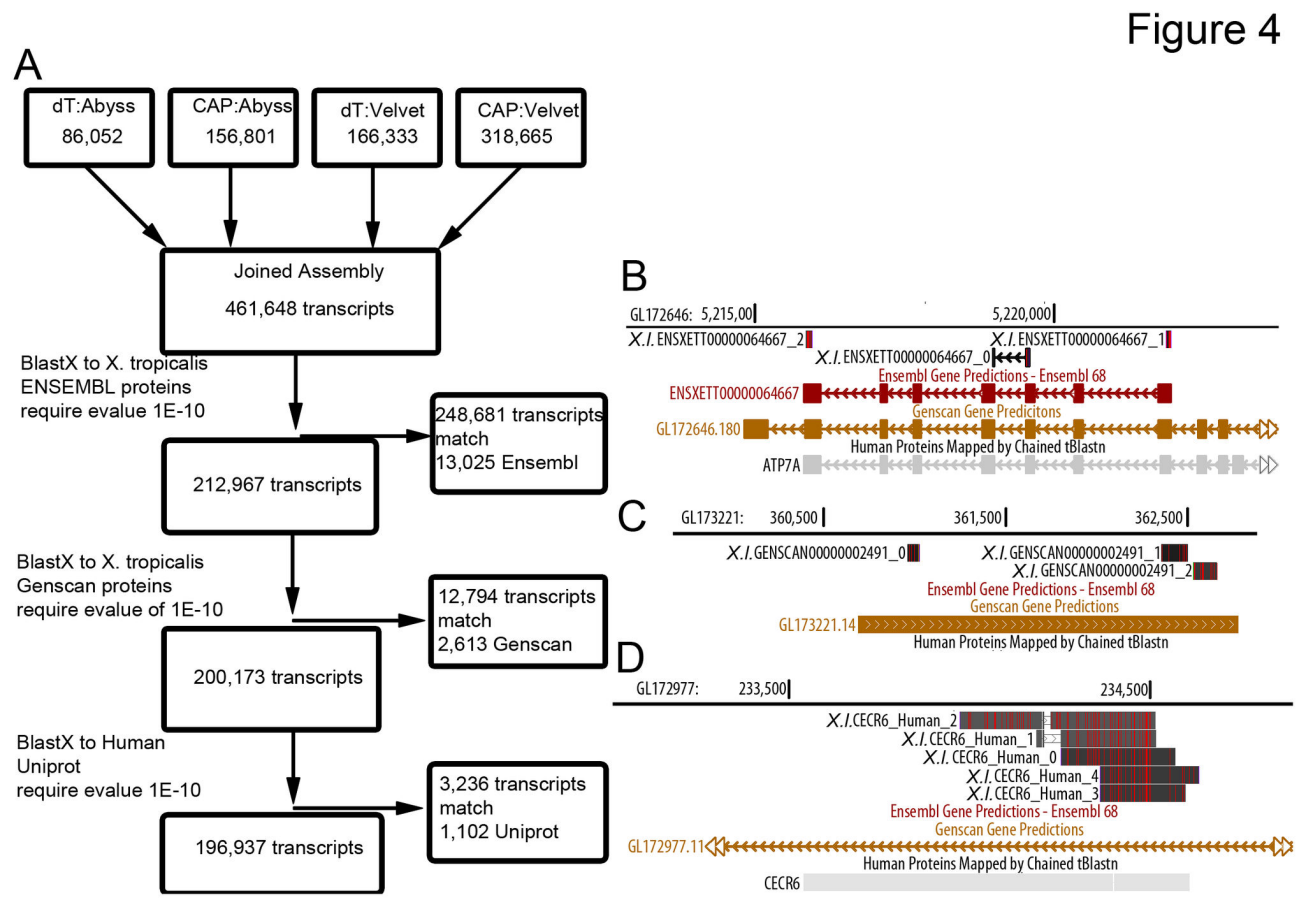
Overview of combined *X. laevis* transcriptome. A. Sequences from each assembler:library pair were combined. Transcripts from this combined library were sequentially aligned to *X. tropicalis* (Xt) ENSEMBL transcripts, *X. tropicalis* Genscan transcripts, and human Uniprot proteins using BLASTX. The number of transcripts that matched to each annotation source and the number of annotated transcripts that were matched are indicated. B. UCSC genome browser view that demonstrates clustering of transcript fragments by using the annotated ENSEMBL transcript as a scaffold. C. UCSC genome browser view of transcript clustering of transcripts that match to a GENSCAN gene prediction. D. Example of several transcripts that match to an annotated human protein while there are no predicted *X. tropicalis* transcripts.

Our results comparing transcripts assembled from different starting input mRNA pools and *de novo* sequence assemblers suggested that each mRNA capture method and assembler identified unique parts of the transcriptome. To generate the most complete transcriptome possible, we combined our four *de novo* assemblies (oligo-dT:Abyss, oligo-dT:Velvet, Cap:Abyss, Cap:Velvet). To remove redundant sequences that were generated by each of the different assembler:library combinations, we combined all of the subassemblies into a single file and used BLAT to find sequences that were entirely contained within another longer sequence as we did for subassemblies generated using different k-mers. After removing redundant sequences we generated a transcript list consisting of 461,648 sequences (Figure 4A). The final assembly consisted of portions of each assembly roughly in proportion to the number of transcripts generated using each approach (Abyss:dT 11%; Cap:Abyss 20%; dT:Velvet 27%; Cap:Velvet 42%). Since *de novo* assemblers generate many short transcripts from rare mRNAs and we noticed that many transcripts map to different regions of the same *X. tropicalis* mRNA, we used a combination of strategies to simplify our transcriptome. First, we used BlastX of transcript sequences to the *X. tropicalis* ENSEMBL annotated proteins as a method to ‘scaffold’ many short transcripts (Figure 4B). Using this approach we are able to determine when short transcripts that are not joined by *de novo* assemblers arise from the same transcript. Additionally, this scaffolding step allowed us to assign more meaningful names to the transcripts generated in our assemblies since *X. tropicalis* ENSEMBL genes are well annotated. Using this approach we found that 248,641 (54%) sequences matched to 13,025 annotated *X. tropicalis* mRNAs. This compares favorably with the 11,935 *X. tropicalis* mRNAs that have clear homologs present in the *X. laevis* NCBI Unigene database. 

Because the *X. tropicalis* ENSEMBL transcript list is not complete[25] we searched the remaining transcripts against the *X. tropicalis* Genscan gene predictions[37] using BlastX. We identified 12,794 transcripts that matched to 2,613 Genscan gene predictions. Manual inspection of these matches revealed that this class of transcripts was accurately predicted by Genscan, but absent from ENSEMBL gene predictions (Figure 4C). 

Finally, we searched the transcripts remaining after alignment to *X. tropicalis* ENSEMBL and Genscan gene predictions to the Uniprot Human proteome using BlastX; we found 3,236 transcripts that matched to 1,102 human proteins. Manual evaluation of these matches revealed that many of these sequences were present in the *X. tropicalis* genome sequence, but absent from both ENSEMBL and Genscan gene predictions (Figure 4D). However, we found that only 20% of these transcripts matched to the *X. tropicalis* genome with high confidence (data not shown). The remaining transcripts had high confidence matches to human proteins (and in some cases *X. laevis* mRNA sequences). These transcripts are likely mRNAs that are expressed from genes that are absent in the current *X. tropicalis* genome assembly. These results are consistent with a recent study of the *X. tropicalis* transcriptome that demonstrated that there are many expressed sequences that are not currently present in the *X. tropicalis* genome assembly[25].

The scaffolding approach reduces the complexity of the *de novo* transcriptome by approximately half and provides information about orthologous genes. However, when this approach is used on an organism with paralogous genes it will collapse paralogs into a single ‘gene’. When many short transcripts from two different paralogs are present it is not possible to separate these sequences into two distinct ‘genes’ without the use of a genomic sequence or a nearly full-length transcript. To determine how many paralogous transcripts were merged by scaffolding to an orthologous transcript we performed BlastN searches of all the transcripts that mapped to an orthologous transcript against each other and searched for sequences that were ~93% identical at the sequence level. Our scaffolding approach found *de novo* transcripts that mapped to 16,740 orthologous transcripts (from *X. tropicalis* ENSEMBL Genscan, and Human Uniprot) with 13,911 that had at least 2 *de novo* transcripts mapped. Of the 13,911 transcripts, 5,465 did not contain any BlastN matches that were consistent with paralogous genes. This suggests that our scaffolding approach collapses paralogous transcripts into one ‘gene’ for ~60% of the orthologous genes. 

To determine if the joined assembly produced by the combination of library preparation methods and assemblers produces a more complete transcriptome than is currently available for *X. laevis* we aligned sequencing reads from libraries used to generate our assembly to the *de novo* transcriptome. We previously found that 43-58% of reads from these libraries aligned to the *X. laevis* Unigene database and that there was a sizeable disparity between the alignment rate for oligo-dT and Cap-captured prepared libraries (Table 1). In contrast, we found that 81-86% of our sequencing libraries aligned to the *de novo* transcriptome and that the alignment difference between oligo-dT and Cap-captured prepared libraries decreased to 5%. To provide an independent test for whether the *de novo* transcriptome was a better representation of the true transcriptome, we prepared sequencing libraries from a different developmental stage (Stage I-III oocytes) than those used to generate the transcriptome (Mature eggs) using both oligo-dT and Cap capture. When we aligned these reads to *X. laevis* Unigene we also observed a low alignment rate (48-59%) and an 11% lower alignment rate for Cap-captured prepared libraries compared to oligo-dT prepared libraries. Alternatively, we found that 80% of the reads from both of these libraries aligned to the *de novo* transcriptome (Table 1), which is considerably greater than the alignment rate to the *X. laevis* Unigene database. The higher alignment rate of libraries prepared from a different developmental stage than those used to generate the transcriptome demonstrates that our *de novo* assembly is a more comprehensive representation of the *X. laevis* transcriptome than is currently available in public databases.

### Characterization of transcripts without orthologs

**Figure 5:**
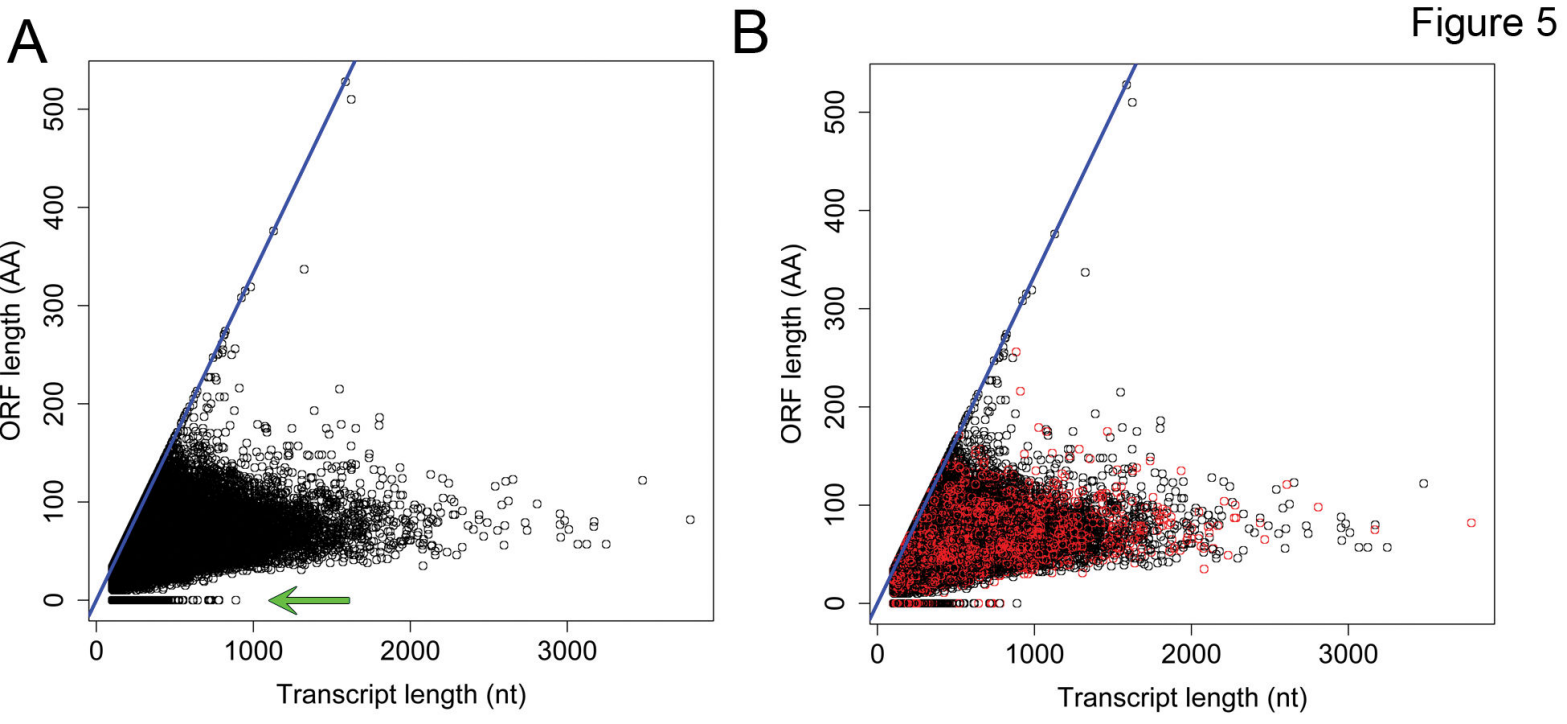
Analysis of unannotated transcripts in *X. laevis*. A. Transcripts remaining after alignment to several sources of annotated genes (in Figure 4A) were analyzed for protein coding potential using Orfpredictor. Plot shows that length of the longest ORF compared to the length of the mRNA sequence, blue diagonal line indicates a transcript that is completely composed of a potential ORF. Horizontal line highlighted with a green arrow indicates sequences with no protein coding potential. B. Same plot as in A, but sequences that matches to annotated *X. laevis* Refseq transcript 3’UTR sequences are highlighted in red.

Recent work studying the transcriptome of many animals has demonstrated that a large fraction of animal genomes is transcribed, but that many of these transcripts lack protein-coding capacity[13-15,38]. Furthermore, work in humans, mice and zebrafish has identified hundreds to thousands of long noncoding RNAs (lncRNAs) that are spliced and polyadenylated, but lack significant protein coding capacity[13-16]. The fact that approximately half of the transcripts that we identified lack clear homologs in the *X. tropicalis* or human proteome suggested that these transcripts could be candidate ncRNAs. To determine if the transcripts with no clear homology to annotated proteins could encode potential ncRNAs we first used ORFPredictor[39] to identify the longest potential open reading frame (ORF) in all 6 reading frames, and found that 98% of these transcripts encoded a potential ORF of any length (mean=43 amino acids) and 27% of the transcripts encoding an ORF of at least 50 amino acids (Figure 5A). One potential source of long transcript fragments with little coding potential are 3’ untranslated regions (UTRs) of mRNAs. To determine if any of the identified transcripts represent transcript fragments that arise from 3’UTRs we used BLASTn to search the *X. laevis* Refseq 3’UTR sequences. We found that 34,000 transcripts (17.3%) had high quality (evalue <= 1e-30) BLASTn matches within the *X. laevis* Refseq 3’UTR sequences. These transcripts included sequences with a range of predicted ORFs and could account for many long sequences with no coding potential (Figure 5B, red points). Given that the *X. laevis* Refseq database only includes 11,054 transcripts, and is not a comprehensive transcriptome, it seems likely that a large fraction of transcripts with little protein coding potential will arise from 3’UTR sequences that could not be linked to the protein coding portion of a transcript during *de novo* assembly. Another potential source of transcripts without homology to known proteins are misassembled transcripts or contaminating transcripts from other organisms (e.g. bacteria or viruses). To determine if transcripts without homology to known proteins have a high portion of misassembled transcripts we mapped all transcripts to a draft assembly of the *X. laevis* genome (Xenbase 6.0). To obtain bona fide matches, we required 95% identity to the genome over the length of the transcript to score a match as positive. We found that approximately 80% of transcripts that had homologs in one of the databases (Ensembl, Genscan or Human Uniprot) matched to the *X. laevis* draft genome. Of the transcripts that did not have homologs in any of the databases, 72% mapped to the genome. As transcripts that do not have protein homologs map to the genome at a rate similar to those that do, we conclude that the transcripts lacking homologs are not likely to be misassembled. At this point we cannot definitively determine if transcripts without homologs are lncRNAs, encode short peptides, or are fragments of longer mRNAs. To definitively determine if these transcripts encode peptides rather than ncRNAs it will be necessary to sequence all transcripts that associate with ribosomes[40]. Further sequencing and transcript assembly will be necessary to determine if these transcripts are unannotated UTR sequences.

## Discussion

Recent advances in DNA sequencing technologies have made it feasible to sequence the entire genome or transcriptome of an organism for a relatively reasonable cost[9]. However, the quality and evenness of coverage of the genome or transcriptome is a function of the input material for library construction. In this current work we have shown that combining different mRNA capture strategies results in a wider coverage of transcribed sequences. This suggests that combining mRNA capture techniques will be a widely applicable method to generate more complete transcriptomes from a range of organisms.

The vast majority of mRNA capture methods are based on using the poly-A tail to select for the mRNA. However, many mRNAs that contain short poly-A tails are inefficiently captured by oligo-dT based methods[17]. A commonly used alternative is to remove rRNA by subtractive hybridization or to use random hexamers that are depleted of sequences that bind to rRNA[18,41,42]. Removal of rRNA is efficient and introduces less bias than selection for poly-A mRNA[18,41]. However, one limitation to this approach is that commercially available rRNA removal kits are tailored for specific organisms and optimization of custom rRNA removal oligos for new organisms can be time consuming and expensive. As another possible alternative to mRNA purification by poly-A selection we explored mRNA capture by the 5’7meG cap. We confirmed previous reports that a mutant form of the human cap-binding protein eIF4E can be used to efficiently capture mRNAs from a pool of total mRNA[21]. By combining Cap-captured mRNAs with oligo-dT captured mRNAs, we were able to generate a more complete transcriptome than either method used alone. Furthermore, the combination of these methods allowed us to identify mRNAs that were undergoing post-transcriptional control through poly-A tail length regulation, which is an important aspect of the normal life cycle of many mRNAs[6,19,43]. This technique should be a widely applicable method to identify new examples of cytoplasmic polyadenylation in many different systems and could be complementary to genome-wide methods that measure the poly-A tail length of mRNAs[8].

Recent advances in *de novo* sequence assemblers that use short sequence reads as input data have allowed the assembly of genomes or transcriptomes from organisms with no genomic resources. Several different *de novo* assemblers have been developed that use similar methodologies, but have different performance characteristics[11]. Applying two of these *de novo* assemblers to our RNA-seq data, we found that each assembler was able to reconstruct similar yet distinct sets of transcripts from the same data. Similar to what we found for different mRNA capture strategies, we found that combining the output from different *de novo* assemblers results in a more comprehensive assembly. It should be tested whether the gain in transcripts can be increased by inclusion of other *de novo* assemblers that were not tested here[15] and if this type of strategy is also applicable to other organisms.

One limitation to the use of *de novo* assemblers is that they tend to generate many short transcripts from genes with low expression levels. In an organism with no sequenced genome it is difficult to know if two short transcripts are part of a larger transcript. To address this issue we have taken the approach of using a closely related organism with a sequenced genome as a scaffold to cluster transcripts. We used annotated genes from *X. tropicalis* to cluster short transcripts from *X. laevis* and found that we could reduce the complexity of our transcriptome by about half. We also found that this approach could be applied by using annotated proteins from a distantly related organism (human). By using multiple organisms as a scaffold we were able to identify a larger number of protein coding genes than would have been possible with either organism alone. Another advantage of this approach is that by using an organism with an annotated genome as a scaffold, we can add meaningful names to the transcripts and link these transcripts to genomic resources available in other organisms with fully sequenced and annotated genomes. This strategy will be useful for organisms that do not (and may never) have a sequenced and annotated genome.

Finally, we found that approximately half of our transcriptome had clear protein-coding homologs in other organisms, and the other half had no clear homologs. The vast majority of these unannotated sequences contained an ORF (albeit a very short one for many sequences). Considering the recent studies that have shown that many putative lncRNAs engage the ribosome and are likely to encode short peptides[44] it is premature to label these transcripts ncRNAs. To definitively assign these RNAs noncoding functions it will be necessary to perform deep sequencing experiments on purified polysomes. 

Taken together we present a general strategy for the capture, sequencing, assembly and annotation of a transcriptome from an organism with an unannotated genome or no available genome sequence. We believe that the approach we have described here using many available software platforms will be applicable to all organisms, and will provide a modest-cost approach to discover expressed genes in any organism.

## Materials and Methods

### DNA constructs and protein expression

eIF4E was amplified from HeLa cell cDNA using the primers: (F) GAATTCATGGCGACTGTCGAACCGGAAA, (R) CTCGAGTTAAACAACAAACCTATTTTTAGTG. eIF4E was subcloned into a pET30a vector containing GFP or PGEX. The mutation K119A[20] was introduced in both constructs by site directed mutagenesis and verified by sequencing. These subclonings generated His-S-GFP-eIF4EK119A (pMB628) or GST-eIF4EK119A (pMB627).

eIF4E expression constructs were transformed into BL21 Rosetta cells. A single colony was used to inoculate a 50ml overnight culture. The following morning this culture was used to inoculate 1L of LB. Cells were grown to an OD of 0.4-0.6. The temperature was shifted to 18°C and IPTG was added to 0.1mM. Cells were grown for an additional 16 hours after induction. Cells were collected by centrifugation and resuspended in PBS + 10mM Imadazole (GFP-eIF4E) or PBS (GST-eIF4E) plus leupeptin, pepstatin, chymostatin and PMSF. Cells were lysed by one passage through a French Press (1200Psi) and the lysate was clarified by centrifugation 25,000 X g for 20 minutes at 4°C. Clarified lysate was incubated with 1ml of NiNTA (Qiagen) or glutathione-sepharose (GE) for 1 hour at 4°C. Beads were washed with 50-100 mls of lysis buffer and eluted with imidazole or reduced glutathione. Typical yield for each construct was ~15-20 mg of protein per liter of culture. Proteins were dialyzed into PBS + 10% glycerol and stored at -80.

### RNA purification

Total RNA was purified from CSF-arrested (mitotic) and interphase *Xenopus laevis* egg extracts and stage I-III oocytes using Trizol (Invitrogen) according to the manufacturers instructions. RNA was resuspended in water.

#### Oligo dT Purificaiton

A total of 5μg of total RNA was used as input for oligo-dT purification using the Illumina tru-Seq mRNA library purification kit for the following libraries: Mitosis Total, Interphase Total, Oocyte dT, Mitosis BWR4, Mitosis Total1, Mitosis Total 2, Taxol Mitosis, SN Mitosis. To compare mRNA capture efficiency between cap-capture and oligo-dT mRNA was purified from 50μg of total RNA using the Exiquon LNA dT purification oligo according to the manufacturers instructions. mRNA yield from the Exiquon kit was ~3-5% of total RNA.

#### 5’Cap capture of mRNA using rEIF4E

5’7meG capped mRNA was purified using a modification of a previously published method[20,21]. 50μg of total RNA was heated to 70°C for 10 minutes then placed on ice. Denatured RNA was diluted into 250μL of buffer A (Buffer A (1×): 10 mM potassium phosphate buffer, pH 8.0, 100 mM KCl, 2 mM EDTA, 5% glycerol, 0.005% Triton X-100, and 1.3% poly(vinyl) alcohol 98–99% hydrolyzed) and 1μL of RNase Inhibitor. 100μg of GFP-eIF4eK119A was added to the RNA and incubated on ice for 30 minutes. GFP-EIF4E was captured by incubation of eIF4E:RNA solution with 200μL of GFP-Trap beads (Allele Biotech), which had been pre-equilibrated in Buffer A, for 30 minutes on ice. Beads were washed 5 times with 1 ml of ice-cold buffer A over the course of ~10 minutes. Beads were switched to new tubes after washes 2 and 4. RNA was eluted and purified form the beads by the addition of either Trizol or solution RLT (from Qiagen RNeasy mini kit). This purification procedure typically yielded ~3-5% of the input RNA (indistinguishable from Exiquon dT purified mRNA) and resulted in a ~10X enrichment of mRNA compared to the starting material. We compared the efficiency and purity of capped RNAs purified using both GST- and GFP- tagged eIF4E. We found that GFP-tagged eIF4E consistently gave both higher yield and purify than GST-eIF4E. We think that this is likely due to the ability to use magnetic beads during the separation procedure for GFP-tagged proteins compared to sepharose beads for GST-tagged proteins. For preparation of oocyte cap-capture libraries mRNA was purified according to the preceding method using 5μg of total RNA. The resulting libraries resulted in similar mRNA enrichment and rRNA depletion as those prepared from a larger scale purification, suggesting that this method can be effectively scaled down for samples with limited input RNA.

### Poly-A tail analysis

Poly-A tail length was estimated using the ePAT method[31]. 1μg of total RNA from either Mitotic or Interphase extract was used as input for the ePAT reaction using the anchor primer (CAAGCAGAAGACGGCATACGATTTTTTTTTTTTTTTTTT). Control reactions were performed by reverse transcription using the following RT primer (CAAGCAGAAGACGGCATACGATTTTTTTTTTTTTTTTTTVN). ePAT or TVN reverse transcriptions reactions were used as input for PCR reactions using the following oligo (CAAGCAGAAGACGGCATACGA) in combination with a gene specific primer. PCR for candidate genes was performed using the following forward primers: (Eg2: CGCTACTTGAATACTGAGTGAATG, Esco2: TTTGGGCTGGAGCTGATTG, (fbox5 CCTTTTTGGGCAACGTTTTG), (stx11 TTTAACCCTTATTTGCTCACATG), (march7 TAGCTGGATGTCCCTTCAAA), (hexim TGTTATGATTTCTTTGTCGGTTTG), (setd8 TCTCAATGGGTTTGCTGCAA), and (MGC83922 TAATGGGCTTTTCATGCATTTCAC). Samples were amplified using a touchdown PCR protocol. Initial annealing temperature was 70°C. The annealing temperature was decreased by 1°C each cycle for 15 cycles, followed by 15 cycles of PCR with an annealing temperature of 55°C. PCR reactions were electrophoresed on a 5% native acrylamide gel, stained with Sybr Gold (Invitrogen) and imaged using a Typhoon imager. For each extract PCR reactions were performed with different cycle numbers (40, 35, 30, 25 total cycles) and the samples where PCR product first appeared were analyzed in order to ensure that the PCR was in the linear range of amplification.

Semi-quantitative assessment of the levels of the above mRNAs was made from the same extracts by performing reverse transcription using random hexamers as RT primers. Each mRNA was amplified from a 5-fold dilution series of the reverse transcription reaction using the following primers: fbox5 (TGCTCCGTTATCTACGTTTTAGT, CCCAATGAGAAAAGCAATTCC), esco2 (TAGTCGCCCCAAAGGAGATT, CTGGCACAATTGTTCCATGA), sttx11 (GTGACCCAGCAACCAGTTTTTT, CTGTATATTGCTTTGCATGTGAGC), march7 (CCAATGTTTTTTTGATTCGACCTG, ACATCAATAGGTCAGTGTTGAAAGT), hexim1 (TTTGTAGCCGGACCCATTAGG, TAAACTCTGGAGGCCTAGCATA), MGC83922 (GCAGTGGTTTGAAAGAAAGACTG, GTGAAATGCATGAAAAGCCC), setd8 (GGAAAAATGCCATATTAAGCTTCC, TTCTCATGTCAAGCCCTATTGT), aurora-a (GCTTATTGACTCAAACACAGGGC, CCGTATATTACAGCATTCAGTAGAG). PCR was performed for 30 cycles and separated on an agarose gel. PCR cycle number was optimized for TVN-RT reactions as described for the ePAT PCR reactions.

### Sequencing and analysis

#### Library construction and sequencing

Libraries were prepared using the Illumina Tru-Seq kit according to manufacturers instructions. This kit uses random hexamer primers and produces unstranded libraries. All Libraries were barcoded and sequenced using an Illumina HiSeq sequencer at the MGH Molecular Biology core facility. All libraries sequenced were single-end 50nt reads. All sequences (from a total of 13 sequencing libraries) have been deposited in the NCBI Short Read Archive (Biopoject Accession # PRJNA191571). Raw sequence files are also available from Xenbase (ftp://xenbaseturbofrog.org/sequence_information/blower_et_al_2013/)

#### Sequence alignment

Sequencing reads were filtered to remove low quality reads. Duplicate reads were removed. We assumed that all identical reads were the result of PCR duplication and do not account for the number of times that a duplicate read was sequenced. All sequences aligning to *Xenopus laevis* rRNA were counted and removed. All remaining reads were aligned to NCBI *Xenopus laeivs* Unigene database (downloaded May 2012) using Bowtie allowing for 2 mismatches per read. Reads per transcript and normalized (reads per Kb per million mapped) were calculated using a custom Perl script.

#### De novo assemblies

17 million unique, non-rRNA reads from libraries generated by oligo-dT capture and Cap-capture were used as input for the *de novo* sequence assemblers Abyss and Velvet. Velvet assemblies were performed for k odd k-values from 17-41. Abyss assemblies were performed for all even k values from 20-40. For each mRNA sample type the resulting transcripts were renamed and merged into a single file. BLAT was used to find sequences that perfectly matched to other sequences within these merged assemblies. Contigs that were completely contained within a longer sequence from the same assembly were removed from the merged assembly.

#### Homology assessment

All sequences from the above *de novo* assemblies were searched against the *Xenopus tropicalis* ENSEMBL transcriptome. Sequences were considered as being a positive hit if they were at least 80% identical to a *X. tropicalis* sequence and the BLAT match covered 50% of the *de novo* transcript length. The number of *de novo* transcripts matching to each *X. tropicalis* transcript and percent coverage of each *X. tropicalis* transcript were calculated using a custom Perl script.

#### Transcript scaffolding

In order to reduce the complexity of the *de novo* sequence assemblies transcripts were scaffolded using a variety of sources (*X. tropicalis* ENSEMBL transcripts, *X. tropicalis* Genscan transcripts, and Human Uniprot proteins). The basic procedure was to use BlastX to find sequences homologous to transcripts from the *de novo* assemblies (requiring an evalue of 1E-10). Transcripts homologous to the same transcript were renamed and numbered according to their closest homolog. Sources used for scaffolding were: *X. tropicalis* ENSEMBL transcripts, *X. tropicalis* Genscan transcript predictions, and Uniprot human proteins. The scaffolding procedure reduced the complexity of the de novo assemblies approximately in half. A FASTA file containing the assembled transcripts is available at Xenbase (ftp://xenbaseturbofrog.org/sequence_information/blower_et_al_2013/)

#### ORF assessment

All transcript sequences remaining after scaffolding were searched for the presence of potential protein coding regions using OrfPredictor[39] and for homology to *X. laevis* Refseq transcript 3’UTRs using BlastN. 

#### Mapping transcripts to *Xenopus laeivs* draft genome

Transcripts were mapped to the 6.0 draft assembly of the *Xenopus laevis* genome (downloaded from Xenbase) using Blat. Alignment to the *X. laevis* genome required 95% identity and a maximum intron size of 20000bp. All mappings were filtered to remove sequences that mapped to more than 2 places in the genome. Mappings were also filtered to remove all alignments that did not cover 80% of the transcript or have a BLAT score of at least 1000. 

### Ethics Statement

All animal work was performed according to standards of animals care and approved by MGH IACUC (OLAW Assurance #: A3596-01). All animal work performed in this study was approved by the Massachusetts General Hospital Subcommittee on Research Animal Care. Frogs were housed in Aquatic Habitats recirculating water housing systems. Water was maintained at a conductivity of ~1800μS and a pH between 7.5-8. Animals were fed frog brittle (Nasco). Frogs were provided with PVC tubes and plastic lilly pads as enrichment. Frogs are handled minimally and all injections are performed using the best possible practices to minimize distress during handling.

## Supporting Information

Figure S1 **Assessment of mRNA enrichment by oligo-dT and cap-capture.** mRNA was captured from total extract using either oligo-dT purification or cap-capture purification. Equal amounts of purified RNA from total extract, dT, or cap-capture was used as input for a reverse transcription reaction using random hexamers as the primer. PCR was performed for vps20 on log dilutions of each reverse transcription reaction to estimate enrichment of mRNA in the purified samples. 200ng of total RNA of purified RNA was used as input for each RT reaction. In the case of dT and cap purified mRNAs this represented ~20% of the total recovered mRNA.
(TIF)

Figure S2 **Biological replicate Poly-A tail analysis of selected mRNAs.** mRNAs that exhibited changes in Mitosis:Interphase (M:IF) abundance ratios in oligo-dT-captured samples, but not in Cap-captured samples were analyzed for poly-A tail length using the ePAT assay and anchored TVN reverse transcription controls. A. Six mRNAs with high M:IF ratios (aurora-a, esco2, fbox5, stx11, march7, and hexim1) showed longer poly-A tails in mitotic extract. Two mRNA (setd8 and MGC83922) with a low M:IF ratio showed very modest changes in poly-A tail lengths between Mitosis and Interphase. B. In addition five mRNAs with high M:IF ratios (aurora-1, fbox5, stx11, march7, and hexim1) had increased amounts of minimal poly-A tail PCR products in mitosis compared to interphase in TVN controls (quantified below gel) while both setd8 and MGC88922 had higher levels of TVN PCR products in interphase compared to mitotic extracts. Ratio of the amount of PCR product in the TVN control lanes is presented below the gel lanes. Ratio of M:IF in dT sequencing libraries is presented below TVN quantification. TVN PCR products indicate mRNAs with poly-A tails of 16 As. C. Semi-quantitative PCR for each of the mRNAs tested in A was performed on RNA from mitotic and interphase extracts. Random hexamers were used to prime reverse transcription for these reactions.
(TIF)

Figure S3 **Comparison of transcript assembly using increased read numbers.** Transcripts were assembled using 17M unique reads from cap-capture libraries (from Figure 3) or 44M unique reads from dT libraries. Transcripts were aligned to *X. tropicalis* ENSEMBL proteins using BlastX (as in Figure 3) and overlap between transcripts sets was calculated. Coverage of each matched transcript is also presented.
(TIF)

Table S1 **Summary of RNA-seq libraries.** IgG samples were immunodepleted using nonspecific rabbit IgG prior to RNA purification. XendoU samples were immunodepleted of XendoU (NP_001128550.1) prior to RNA purification. Taxol Mitosis samples were mitotic extract incubated with 10μM taxol for 30 minutes prior to RNA purification. SN Mitosis was mitotic extract incubated with sperm nuclear DNA for 30 minutes prior to RNA purification. BWR4 Mitosis .
(DOCX)

Table S2 **mRNAs with high and low M:IF ratios in dT RNA-seq libraries.**
(TXT)

Table S3 **mRNAs with high and low M:IF ratios in dT RNA-seq libraries.**
(TXT)
