# Supplementary figures and images for: Combining Different mRNA Capture Methods to Analyze the Transcriptome: Analysis of the Xenopus laevis Transcriptome
Source: PLoS One. 2013 Oct 15;8(10):e77700. doi: 10.1371/journal.pone.0077700 (PMC3797054; doi:10.1371/journal.pone.0077700)

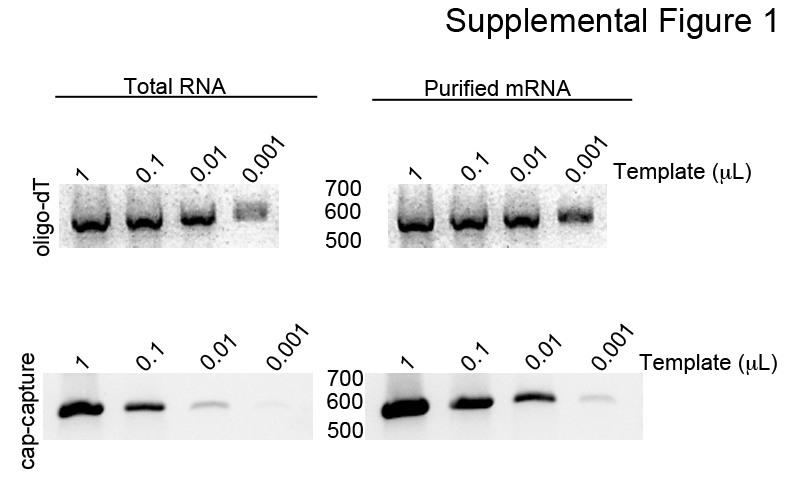

Supplement: Figure S1 — Assessment of mRNA enrichment by oligo-dT and cap-capture. mRNA was captured from total extract using either oligo-dT purification or cap-capture purification. Equal amounts of purified RNA from total extract, dT, or cap-capture was used as input for a reverse transcription reaction using random hexamers as the primer. PCR was performed for vps20 on log dilutions of each reverse transcription reaction to estimate enrichment of mRNA in the purified samples. 200ng of total RNA of purified RNA was used as input for each RT reaction. In the case of dT and cap purified mRNAs this represented ~20% of the total recovered mRNA. (TIF) [file pone.0077700.s001.tif]

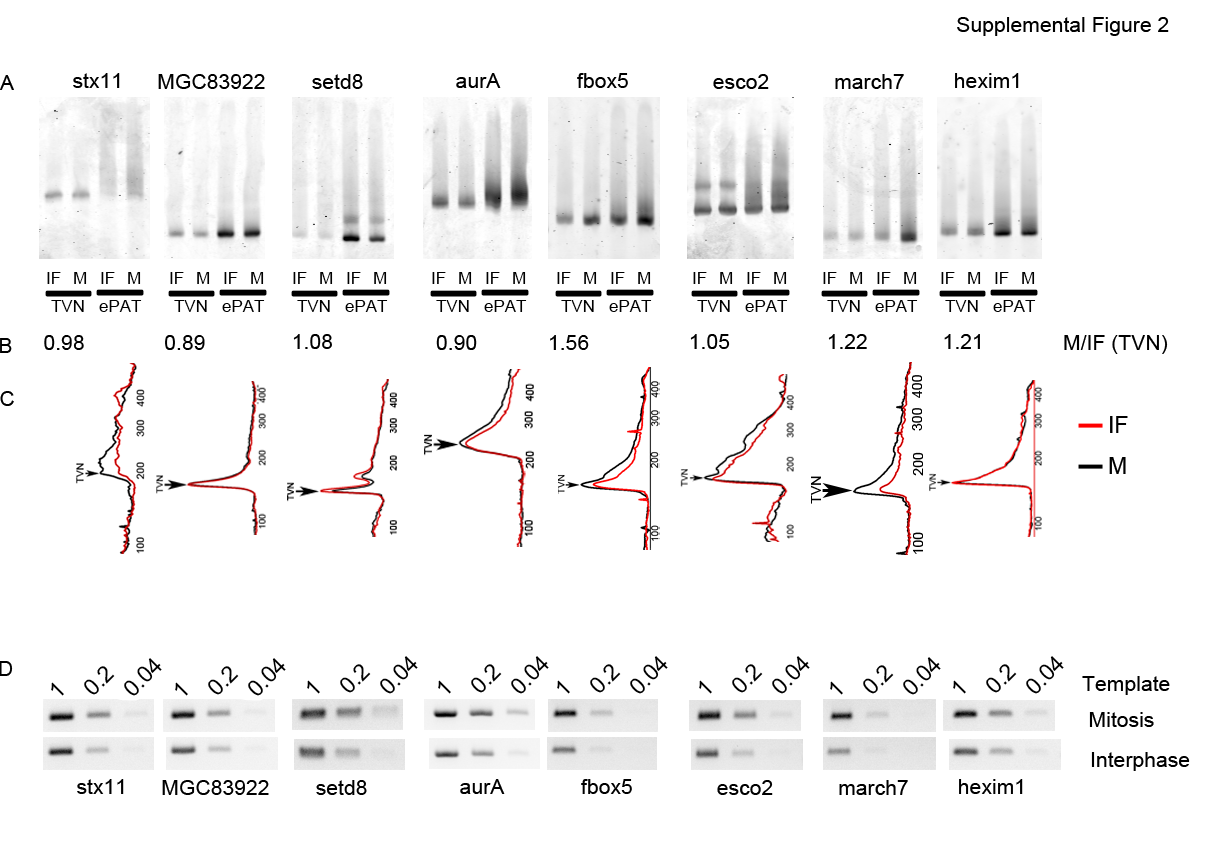

Supplement: Figure S2 — Biological replicate Poly-A tail analysis of selected mRNAs. mRNAs that exhibited changes in Mitosis:Interphase (M:IF) abundance ratios in oligo-dT-captured samples, but not in Cap-captured samples were analyzed for poly-A tail length using the ePAT assay and anchored TVN reverse transcription controls. A. Six mRNAs with high M:IF ratios (aurora-a, esco2, fbox5, stx11, march7, and hexim1) showed longer poly-A tails in mitotic extract. Two mRNA (setd8 and MGC83922) with a low M:IF ratio showed very modest changes in poly-A tail lengths between Mitosis and Interphase. B. In addition five mRNAs with high M:IF ratios (aurora-1, fbox5, stx11, march7, and hexim1) had increased amounts of minimal poly-A tail PCR products in mitosis compared to interphase in TVN controls (quantified below gel) while both setd8 and MGC88922 had higher levels of TVN PCR products in interphase compared to mitotic extracts. Ratio of the amount of PCR product in the TVN control lanes is presented below the gel lanes. Ratio of M:IF in dT sequencing libraries is presented below TVN quantification. TVN PCR products indicate mRNAs with poly-A tails of 16 As. C. Semi-quantitative PCR for each of the mRNAs tested in A was performed on RNA from mitotic and interphase extracts. Random hexamers were used to prime reverse transcription for these reactions. (TIF) [file pone.0077700.s002.tif]

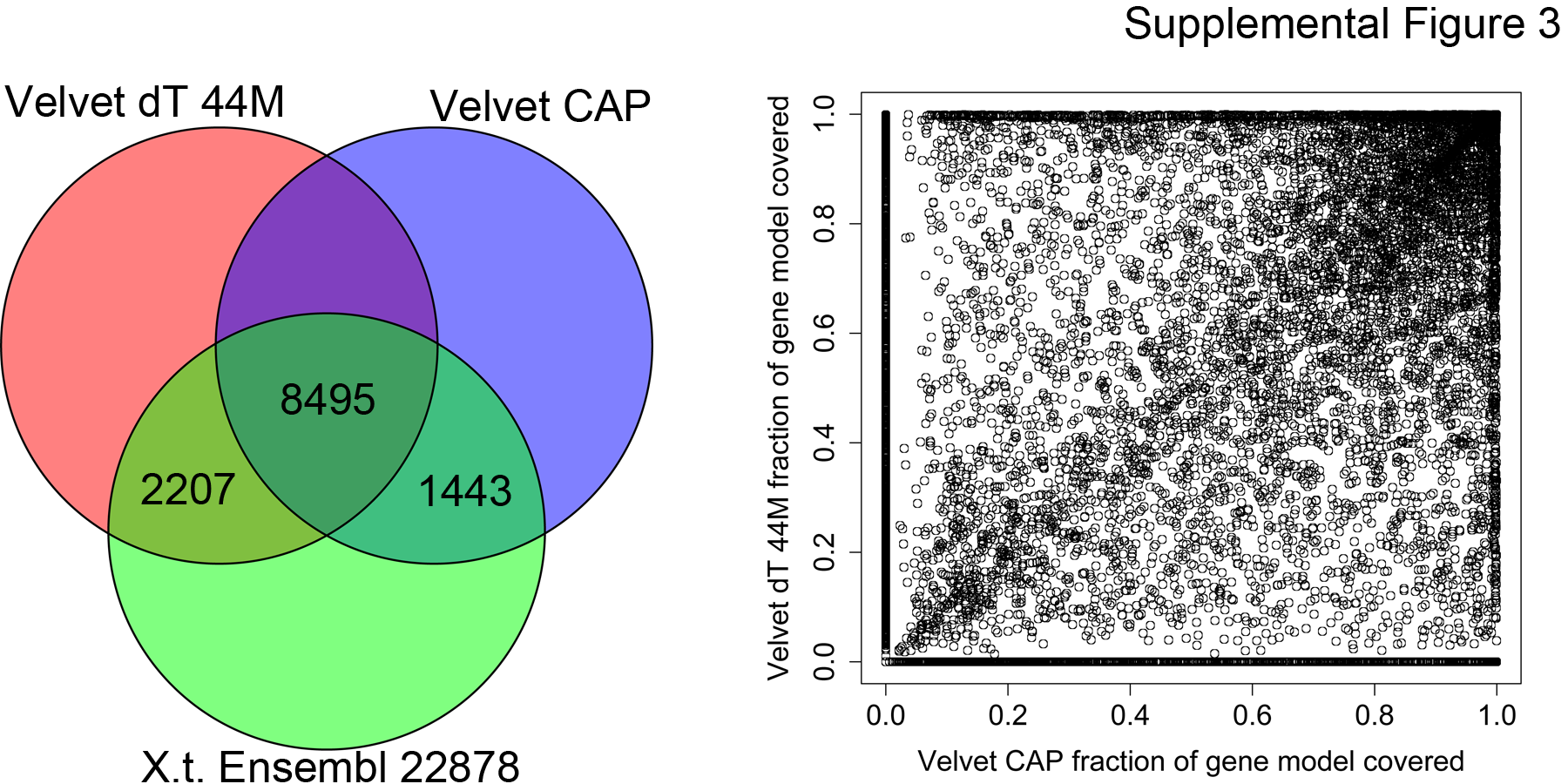

Supplement: Figure S3 — Comparison of transcript assembly using increased read numbers. Transcripts were assembled using 17M unique reads from cap-capture libraries (from Figure 3) or 44M unique reads from dT libraries. Transcripts were aligned to X. tropicalis ENSEMBL proteins using BlastX (as in Figure 3) and overlap between transcripts sets was calculated. Coverage of each matched transcript is also presented. (TIF) [file pone.0077700.s003.tif]
