# Supplementary material for: Combining Different mRNA Capture Methods to Analyze the Transcriptome: Analysis of the Xenopus laevis Transcriptome
Source: PLoS One. 2013 Oct 15;8(10):e77700. doi: 10.1371/journal.pone.0077700 (PMC3797054; doi:10.1371/journal.pone.0077700)
Supplement: Table S1 — Summary of RNA-seq libraries. IgG samples were immunodepleted using nonspecific rabbit IgG prior to RNA purification. XendoU samples were immunodepleted of XendoU (NP_001128550.1) prior to RNA purification. Taxol Mitosis samples were mitotic extract incubated with 10μM taxol for 30 minutes prior to RNA purification. SN Mitosis was mitotic extract incubated with sperm nuclear DNA for 30 minutes prior to RNA purification. BWR4 Mitosis . (DOCX) [file pone.0077700.s004.docx]

| **Library** | **mRNA capture** | **reads** | **unique reads** | **%rRNA** | **alignment to Unigene** |
| --- | --- | --- | --- | --- | --- |
| **Mitosis Total** | dT | 21877083 | 8587598 | 1.86 | 57.2 |
| **Interphase Total** | dT | 24749671 | 11764346 | 1.03 | 57.93 |
| **IgG Mitosis** | CAP | 36117887 | 9565509 | 2.1 | 43.04 |
| **IgG Interphase** | CAP | 28443149 | 7558752 | 2.2 | 43.26 |
| **XendoU Mitosis** | CAP | 57936155 | 17740053 | 1.39 | 43.29 |
| **XendoU Interphase** | CAP | 60291921 | 17947155 | 1.45 | 42.98 |
| **Oocyte** | dT | 29272426 | 16037630 | 0.02 | 58.6 |
| **Oocyte** | CAP | 46621901 | 15118718 | 0.9 | 47.88 |
| **Mitosis BWR4** | dT | 63886487 | 23854519 | 0.93 | 54.47 |
| **Mitosis Total 1** | dT | 40942363 | 16599958 | 1.01 | 56.27 |
| **Mitosis Total 2** | dT | 31845008 | 12823829 | 1.2 | 57.19 |
| **Taxol Mitosis** | dT | 30137771 | 11359244 | 1.87 | 54.23 |
| **SN Mitosis** | dT | 28571200 | 10181967 | 2.2 | 53.87 |

**Supplemental Table 1. Libraries sequenced and characteristics.**
